# Capturing Human Perceptual and Cognitive Activities via Event-Related Potentials Measured with Candle-Like Dry Microneedle Electrodes

**DOI:** 10.3390/mi11060556

**Published:** 2020-05-30

**Authors:** Yuri Yoshida, Takumi Kawana, Eiichi Hoshino, Yasuyo Minagawa, Norihisa Miki

**Affiliations:** 1 Department of Mechanical Engineering, Keio University, 3-14-1 Hiyoshi, Kohoku-ku, Yokohama, Kanagawa 223-8522, Japan; yurikenwanwanwan@gmail.com (Y.Y.); taku-23mp@keio.jp (T.K.); 2 Department of Psychology, Keio University, 4-1-1 Hiyoshi, Kohoku-ku, Yokohama, Kanagawa 223-8521, Japan; eiichi.hoshino.c@gmail.com (E.H.); myasuyo@bea.hi-ho.ne.jp (Y.M.)

**Keywords:** electroencephalogram, event-related potential, candle like, dry electrodes, microneedle electrodes, oddball task, near-infrared spectroscopy, P300, MMN, laterality

## Abstract

We demonstrate capture of event-related potentials (ERPs) using candle-like dry microneedle electrodes (CMEs). CMEs can record an electroencephalogram (EEG) even from hairy areas without any skin preparation, unlike conventional wet electrodes. In our previous research, we experimentally verified that CMEs can measure the spontaneous potential of EEG from the hairy occipital region without preparation with a signal-to-noise ratio as good as that of the conventional wet electrodes which require skin preparation. However, these results were based on frequency-based signals, which are relatively robust compared to noise contamination, and whether CMEs are sufficiently sensitive to capture finer signals remained unclear. Here, we first experimentally verified that CMEs can extract ERPs as good as conventional wet electrodes without preparation. In the auditory oddball tasks using pure tones, P300, which represent ERPs, was extracted with a signal-to-noise ratio as good as that of conventional wet electrodes. CMEs successfully captured perceptual activities. Then, we attempted to investigate cerebral cognitive activity using ERPs. In processing the vowel and prosody in auditory stimuli such as /itta/, /itte/, and /itta?/, laterality was observed that originated from the locations responsible for the process in near-infrared spectroscopy (NIRS) and magnetoencephalography experiments. We simultaneously measured ERPs with CMEs and NIRS in the oddball tasks using the three words. Laterality appeared in NIRS for six of 10 participants, although laterality was not clearly shown in the results, suggesting that EEGs have a limitation of poor spatial resolution. On the other hand, successful capturing of MMN and P300 using CMEs that do not require skin preparation may be readily applicable for real-time applications of human perceptual activities.

## 1. Introduction

To investigate brain activities non-invasively, a variety of measurement methods have been developed, including functional magnetic resonance imaging (fMRI), magnetoencephalography (MEG), near-infrared spectroscopy (NIRS), and electroencephalography (EEG) [1,2,3,4]. Although functional magnetic resonance imaging and MEG require bulky systems and request the participants to maintain their head and body positions, NIRS and EEG involve rather simple and light systems and require less restriction of participants. Therefore, NIRS and EEG are suitable to investigate the brain activity utilized in daily life applications. NIRS has superior spatial resolution, whereas EEGs have more temporal resolution and are more suitable for real-time applications [5,6].

A great challenge in applying EEG is the attachment of the electrodes. Such a barrier that makes participants hesitate to receive the beneficial information from an EEG must be overcome. The preparation requisite for measurement is preferred to be least. Therefore, wet electrodes that require time-consuming skin treatment and conductive glue are not good candidates. Avoiding hairs when attaching electrodes, which may prevent a good interface between the electrodes and the head, is also challenging. All measurement points except the frontal region are located on hairy regions. Characteristic brain waves can be acquired from measurement sites other than the frontal region, such as the parietal and occipital regions involving Cz and Pz in the international 10–20 system [7,8]. Therefore, a measurement system that can measure brain activities from hairy regions with minimal preparation is mandatory for routine studies of brain activities.

**Figure 1:**
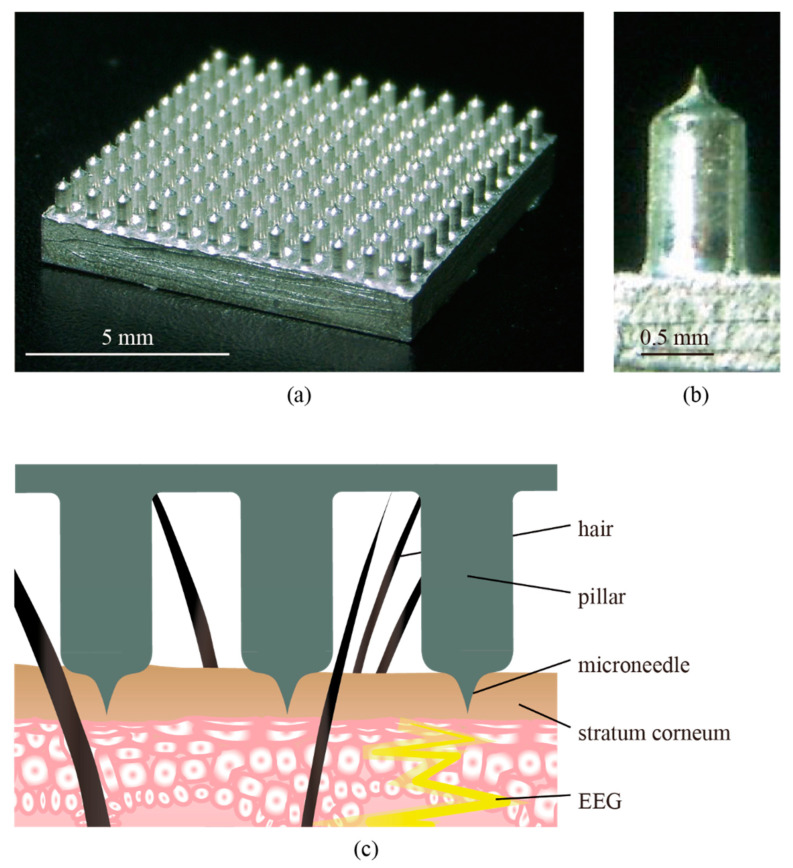
(**a**,**b**) Photos and (**c**) the concept of the candle-like microneedle electrodes (CMEs). The roots avoid hairs, and the needle on the tip penetrates through the high-impedance stratum corneum. Note that all the needle electrodes are electrically connected. The fabrication process is detailed in our prior work [17].

Therefore, dry electrodes that can acquire EEG from hairy regions have been developed using unique materials, microfabrication techniques, and attachment mechanisms [9,10,11,12,13,14,15,16]. We developed candle-like dry microneedle electrodes (CMEs), as shown in Figure 1 [17]. The electrodes can measure EEG from hairy regions without any preparation. The roots of the electrodes avoid hairs, and the tips penetrate through the stratum corneum, which sufficiently reduces skin–electrode contact impedance. The needles are designed not to reach the pain point, and CMEs inflict no pain on participants. In our previous work, we successfully measured spontaneous potentials of EEG from occipital regions using CMEs [17]. Although the measurements did not require abrasion of the stratum corneum or conductive gels, the results were comparable to those from the conventional wet electrodes with skin preparation. In addition, we successfully extracted indices that represent mental fatigue of the participants [18]. However, these results were based on frequency-based signals, which are relatively robust compared to noise contamination, and whether CMEs are sufficiently sensitive to capture finer signals remained unclear.

Consequently, in this work, we aimed to measure the event-related potentials (ERPs) of EEG using CMEs. ERPs are EEG indices, which are electrophysiological responses that reflect perceptual or cognitive processing of stimuli. From ERPs, the specific response of the brain that represents neural processing of each stimulus according to its time order can be deduced. P300 is a major signal of ERP, appears approximately 300 ms after the stimulus onset, and reflects the conscious recognition of the difference in stimuli [19]. Another major component, mismatch negativity (MMN), is an ERP that appears as a negative deflection around 100–150 ms after presentation of the deviant stimulus and reflects the automatic recognition of differences primarily in auditory stimuli [20,21]. Given the high temporal resolution of EEG, detection of these characteristic ERPs can be applied to real-time brain-tech applications. One of the originality and novelty of this work is successful characterization and demonstration of ERP measurement with CMEs.

However, the poor spatial resolution of EEG may pose a limitation. The differences in cognitive processing of the vowel and prosody conditions were captured by MEG and NIRS [22,23,24], and right-handed adult participants show lateralized speech language processing [23]. Vowel processing is predominantly processed in the left auditory area, whereas the right auditory area is chiefly responsible for prosody processing. Therefore, in the oddball tasks using /itta/, /itte/, and /itta?/, NIRS captures an increase in the oxygenated hemoglobin (Oxy-Hb) concentration and decrease in deoxygenated hemoglobin in the left and right auditory area in response to vowel and prosody changes, respectively [22,23]. We investigated whether EEG acquired using CMEs could capture laterality. The other originality and novelty of this work is application of CMEs to neuroscience study.

## 2. Methods

All the experiments in this paper were approved by the research ethics committee of the Faculty of Science and Technology, Keio University.

### 2.1. ERP Measurement with CMEs and Conventional Wet Electrodes

We used CMEs and commercially available wet electrodes (NE-114A, Nihon Kohden, Tokyo, Japan) to measure ERPs. The fabrication process of the CMEs is reported in our prior work [17]. Auditory oddball tasks using pure tones involve presentation of standard stimuli more frequently than deviant stimuli. When the deviant stimulus is presented to the participant, he/she recognizes the difference from previously memorized stimuli, or the standard stimuli, and an ERP is evoked. Due to the small amplitude of ERPs, ERPs are extracted by averaging multiple series of signals after the corresponding deviant stimuli [25]. The number of deviant stimuli required to extract the ERPs represents the effectiveness of the electrodes. Eight healthy men and three healthy women aged 22–25 years participated in the experiments.

#### 2.1.1. Oddball Task

The task consisted of pure tones of 1000 Hz and 2000 Hz. The stimuli were presented at an interval of 1500 ms with a duration of 1000 ms. The tones of 1000 Hz and 2000 Hz were presented 280 and 70 times as the standard stimuli and the deviant stimuli, respectively. The sequences of the standard and deviant stimuli were mixed such that the deviant stimulus was presented after 3–5 standard stimuli in series. To maintain the concentration of the participants, they were requested to press a button every time they heard a deviant during the experiments. The audio samples are available in Supplementary Materials Audio S1.

#### 2.1.2. Measurement

**Figure 2:**
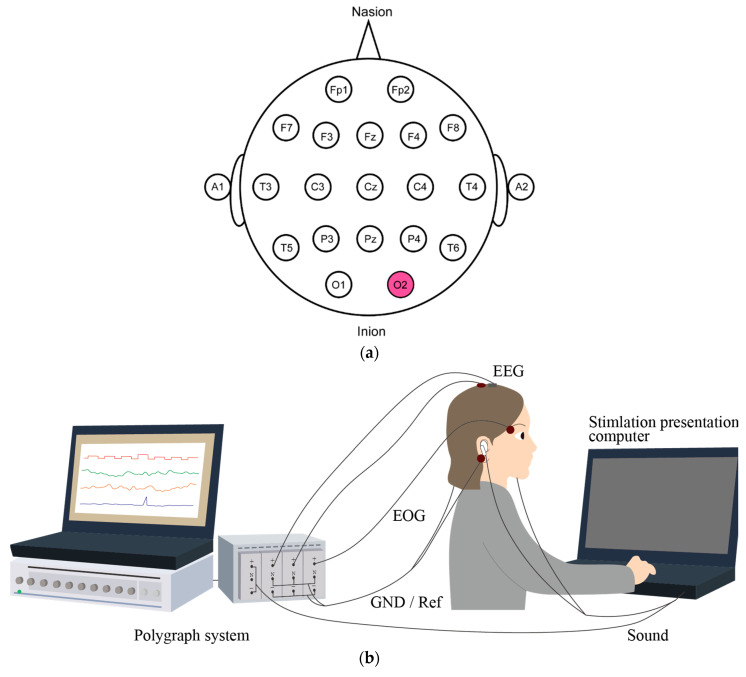
(**a**) Measurement sites of electroencephalogram (EEG) based on the 10–20 system. (**b**) Event-related potentials (ERPs) measurement system. EEG was measured from Cz with the conventional wet electrode and CME simultaneously. Auditory stimuli were presented to the participant. The signals were acquired and processed by the polygraph system.

Figure 2 illustrates the experimental system. EEG signals and electrooculography (EOG) signals were recorded on a polygraph system (RMT-1000, Nihon Kohden) at a sampling rate of 1000 Hz. EEG was measured at Cz in the international 10–20 system (see Figure 2a), at the vertex of the head [26], with the CME and the wet electrode next to each other with a gap smaller than 1 cm simultaneously. EOG was measured at the right supraorbital border with the CME. The reference and ground were measured at A1 and A2 with the wet electrodes, respectively, and they were shorted. No preparation was used for the CME, whereas conventional skin preparation, i.e., abrasion of the stratum corneum and use of conductive paste, was applied for the wet electrodes. These electrodes were fixed with a flexible band.

Auditory stimuli were recorded on the polygraph system in a sound wave form. The start of each stimulus was used as the trigger for averaging of the EEG. EEG and EOG signals were processed through band-pass filters of 0.05–30 Hz and 0.5–10 Hz, respectively, using software (Labchart, ADinstruments, Sydney, Australia).

#### 2.1.3. Analysis

All signals were analyzed with software (MATLAB R2018b, Math Works, Natick, MA, USA). ERPs were extracted from the acquired EEG using the averaging method, which was proposed by Dawson [25]. We collected the EEG data from 100 ms before to 1000 ms after the trigger. We considered one set of data to be a trial. When the amplitude of the EOG exceeded 100 µV and/or the amplitude of the EEG exceeded 50 µV, the trial was excluded from the analysis. ERPs are not elicited when participants have a low consciousness level [27]. When the participants failed to push the button, the trial was also excluded.

After the drift of the EEG signals was removed, averaging was conducted. The number of trials for averaging to observe P300 with a significant difference was measured to assess the effectiveness of the electrodes.

### 2.2. ERP Measurement with CMEs and NIRS

We compared ERPs measured with CMEs to the hemodynamic response measured with NIRS when the participants performed oddball tasks to investigate their cognitive activities. Because ERPs appear during a very short time in EEG, they have the potential to be used in real-time applications.

We presented an oddball task that consists of three speech sounds, which were /itta/, /itte/, and /itta?/, as described in prior work [22,23,24]. These are the past, imperative, and question forms, respectively, of the Japanese verb /iku/ (“go” in English). Recognition of the difference in the vowel and prosody conditions was captured by MEG [24] and NIRS [22,23]. Speech language processing is lateralized in right-handed adults [23]. The vowel in /itta/ and /itte/ is processed in the left auditory area, whereas the right auditory area is responsible for prosody processing (/itta/ and /itta?/). NIRS successfully captured the increase in the Oxy-Hb concentration and the decrease in deoxygenated hemoglobin in the left and right auditory areas for vowel and prosody processing, respectively [22,23].

First, we measured the EEG from the parietal region with CMEs and extracted ERPs during the oddball task. Second, we simultaneously measured ERPs with CMEs and NIRS from the bilateral auditory areas for comparison.

#### 2.2.1. Oddball Task

The tasks consisted of three speech sounds. The length and strength of the sounds were set to be the same. The first syllable was /i/, and the length was 80 ms for all three sounds. After a silent interval of 200 ms, the second syllable started. The length of the second syllable was 92 ms. The second syllables were different among the three sounds; /itte/ is different from /itta/ in the phoneme condition. /itta?/ is different from /itta/ in the prosody condition.

In the first experiment, the sound stimuli were presented at an interval of 1500 ms. /itte/ and /itta?/ were presented 70 times each as the deviant stimuli, and /itta/ was presented 560 times as the standard stimulus. The stimuli were presented at random sequences with the following conditions: one of the two deviant stimuli was presented one time after the standard stimuli were presented 3–5 times in series, and the same deviant stimulus was not presented more than three times in a row. To maintain the concentration of the participants, they were requested to press a button every time they heard a deviant during the experiments.

In the EEG/NIRS experiment, sound stimuli were presented at an interval of 1000 ms. /itte/ and /itta?/ were presented 36 times each as the deviant stimuli, and /itta/ was presented 802 times as the standard stimulus. The stimuli were presented at random sequences with the following conditions: one of the two deviant stimuli was presented one time after the standard stimuli were presented 22–24 times, and the same deviant stimuli were not presented more than three times in a row. To maintain the concentration of the participants, they were requested to press a button every time they heard a deviant during the experiments. 

#### 2.2.2. Measurement

In the first experiment, the experimental setup and signal processing were same as the pure tone experiments. There were 10 participants in total—7 healthy men and 3 healthy women aged 22–25 years.

**Figure 3:**
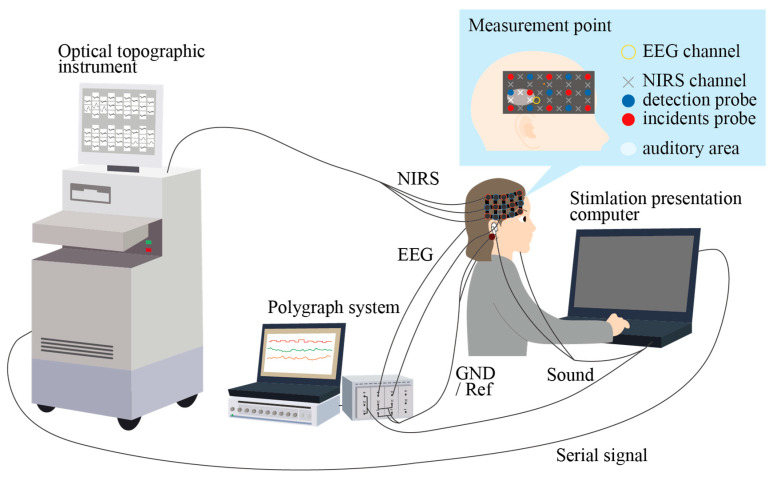
ERP and near-infrared spectroscopy (NIRS) measurement system. EEG was measured at T3 and T4 with CMEs. NIRS signals were measured with eight incidents and seven detection probes, each of which was separated by a 3-cm square lattice. The probes were placed over the measurement region in 3 × 5 lattice shapes on the right and left temporal regions. Three channels were positioned over the auditory area.

In the experiment with simultaneous EEG/NIRS measurement, EEG signals were recorded on a polygraph system (RMT-1000, Nihon Kohden) at a sampling rate of 1000 Hz. EEG was measured at T3 and T4 in the international 10–20 system [26] with CMEs. The reference and ground were obtained with conventional wet electrodes from A1 and A2, respectively, and they were shorted. No preparation was conducted for T3 and T4 EEG measurements with CMEs, and A1 and A2 were obtained after conventional preparation for wet electrodes. CMEs were fixed with flexible bands. The auditory stimuli were recorded on the polygraph system in a sound wave form, and the start time of the second syllable was used as the trigger. EEG signal processing was the same as that of the pure tone experiments. NIRS signals were recorded on an optical topographic instrument (OT-R40, Hitachi Medical Corporation, Tokyo, Japan) at a sampling rate of 10 Hz. Eight incidents and seven detection probes, each of which was separated by a 3-cm square lattice, were placed over the measurement region in 3 × 5 lattice shapes on the right and left temporal regions, as shown in Figure 3. This setup resulted in 22 channels of NIRS measurement. Three channels were located over the auditory area as illustrated by the white region in Figure 3. The serial signals were transmitted to the system at the start of each stimulus, which was used as the trigger. All acquired signals were filtered with software (Labchart, ADinstruments, Sydney, Australia). EEG signals and the concentrations of Oxy-Hb measured by NIRS were processed through band-pass filters of 0.05–30 Hz and 0.01–0.8 Hz, respectively.

#### 2.2.3. Analysis

All signals were analyzed with MATLAB (Math Works). ERPs were extracted from the acquired EEG using the same process as used in the pure tone experiments. The concentration of Oxy-Hb was analyzed with the averaging method to observe the response. We smoothed the data with a 5-s moving average. Data were collected from 5 s before to 15 s after the trigger. These 20-s-long data were considered one trial. When body motion noises severely affected the measurement and the concentration of Oxy-Hb exceeded 3 mM·mm, the trials were excluded. We also excluded trials when the participant failed to push the button. For the remaining trials, we removed linear drift from the NIRS data. The average of the concentrations of Oxy-Hb 5 s before the trigger was calculated and subtracted from the concentrations of Oxy-Hb of each trial. The processed signals were averaged to extract the response to the deviant stimuli for three channels over the auditory area for the left and right temporal regions. The signals that showed the largest change for each region were used as the characteristic response signals.

## 3. Results and Discussion

### 3.1. ERP Measurement with CMEs and Conventional Wet Electrodes

**Figure 4:**
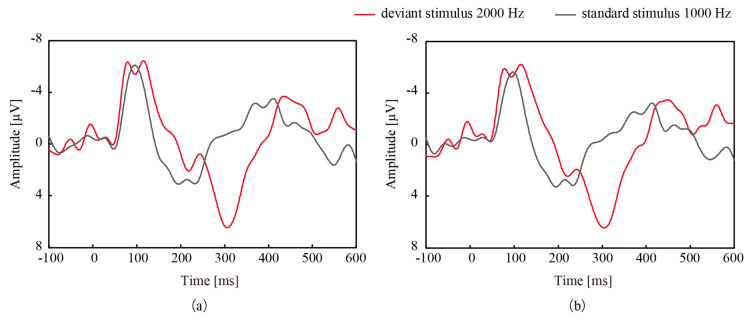
Grand averaged signal measured at Cz from participant 1 acquired with (**a**) CMEs and (**b**) wet electrodes. In both cases, mismatch negativity (MMN) and P300 were observed.

Figure 4 shows the grand averaged signal over the course of an experiment, which consisted of the standard stimuli played 280 times and the deviant stimuli played 70 times, from participant 1. The grand averaged signal acquired with CMEs was similar to that acquired with wet electrodes, and this trend was observed for all participants. A strong correlation was noted between the signals from CMEs and the wet electrodes, as shown in Table 1, even though CMEs do not require any preparation.

**Figure 5:**
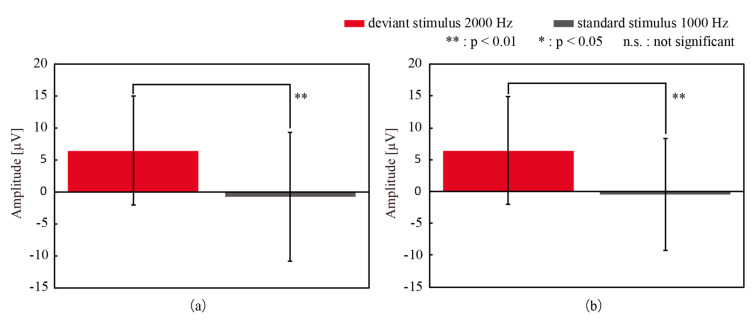
The amplitudes of EEG (**a**) 307 ms after the deviant and standard stimuli with CMEs and (**b**) 305 ms after the stimuli with wet electrodes. A significant difference was found between the response to deviant and standard stimuli. Error bars: standard deviation.

Next, we investigated ERPs acquired with both electrodes. As shown in Figure 4a,b, a positive deflection was found around 300 ms after the deviant stimulus presentation for both types of electrodes. Note that the vertical axes of the figures are drawn upside down from the conventional manner. The amplitude and latency of the deflection with CMEs and the wet electrodes were 6.46 µV and 307 ms and 6.45 µV and 305 ms, respectively. The independent *t*-test was conducted to compare the amplitudes after the latency time of the deviant stimuli and the standard stimuli. Figure 5 shows the average and standard deviation of the amplitudes at 307 ms (CMEs) and 305 ms (wet electrodes) after all deviant and standard stimuli for participant 1. This figure clearly shows the significant differences in both cases. The deflection was considered to be P300 with high likelihood. This P300 was found for all 10 participants when using both electrodes and was statistically significant.

**Figure 6:**
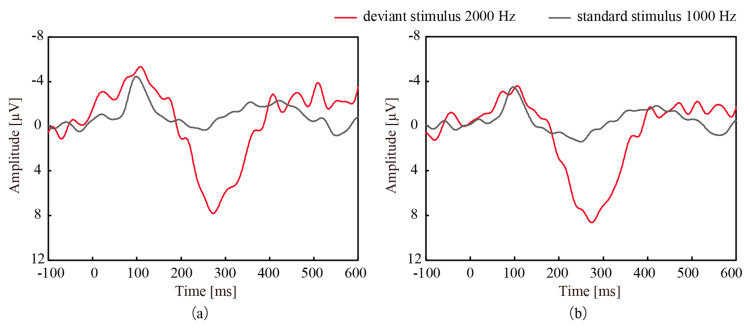
Grand mean waveform measured at Cz from all 10 participants after the deviant and standard stimuli measured with (**a**) CMEs and (**b**) the wet electrodes.

**Figure 7:**
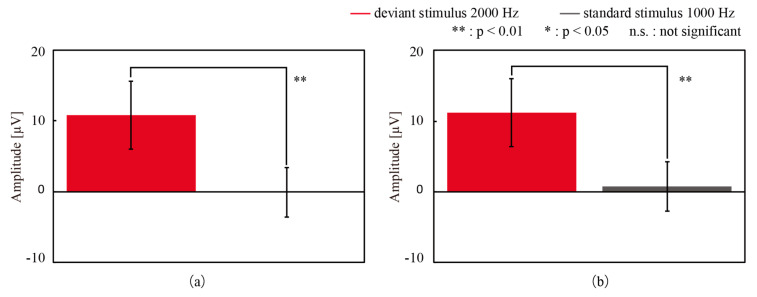
The amplitudes of the grand mean wave after the latency time to the deviant and standard stimuli with (**a**) CMEs and (**b**) the wet electrodes. Significant differences between the stimuli were obtained for both electrodes. Error bars: the standard deviation.

We detected P300 from the grand averaged signals, which were the average of all participants, as shown in Figure 6. A positive deflection was found approximately 300 ms after the deviant stimulus presentation for both electrodes. The amplitude and the latency of the deflection acquired with CMEs and the wet electrodes were 7.81 µV and 274 ms and 8.64 µV and 275 ms, respectively. Figure 7 shows the average and standard deviation of the amplitudes after 274 ms and 275 ms of the latency for all participants, which were considered to be P300. The paired *t*-test was conducted to compare the amplitudes of P300 and the latency for the standard stimuli, and significant differences were found.

**Figure 8:**
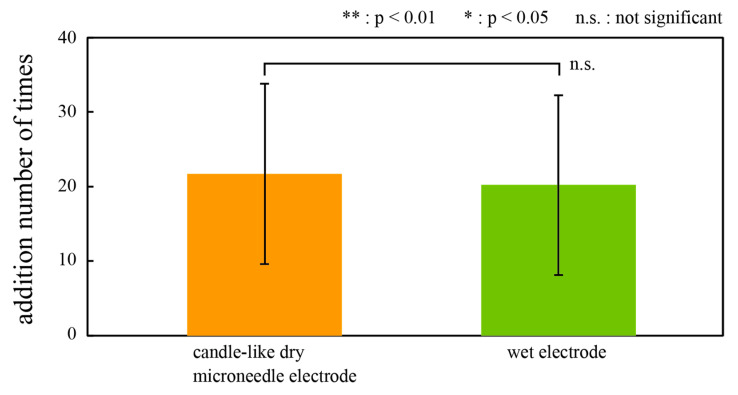
The number of trials required to extract ERPs with statistical significance with the averaging method. CMEs and the wet electrodes showed similar efficiency. Error bars: the standard deviation.

Finally, we investigated the efficiency of CMEs and the wet electrodes to detect P300, which can be assessed by the number of deviant stimuli to deduce P300 with statistical significance with the averaging method. With the averaging method, spontaneous potentials and noise are more efficiently cancelled, and ERPs appear with higher significance as the number of trials increases. More efficient electrodes require fewer trials to extract ERPs. Figure 8 shows the average and standard deviation of the number of stimuli necessary to detect P300 with statistical significance (*p* < 0.05) for all participants. For each number of additions for each trial to a deviant stimulus, a *t*-test was performed using the mean of the amplitude of P300 before and after the apex latency of 5 ms as a sample, and the number of additions until P300 was found to be a statistically significant component was shown.

The results showed no significant difference between CMEs and the wet electrodes. From these results, we conclude that CMEs that require no skin preparation can acquire ERPs from all participants with accuracy as good as conventional wet electrodes. Given that the wet electrodes are currently considered to be the best way to obtain ERPs even though they require time-consuming and uncomfortable skin preparation, the proposed CMEs may be a better alternative for accurate and efficient ERP measurement. P300 is the response to the experience of unexpected stimuli and corresponds to perceptual activities. Efficient capture of P300 may be effective for brain-tech applications.

### 3.2. Correlation Between ERPs Measured with CMEs and NIRS

#### 3.2.1. Preliminary Experiments

**Figure 9:**
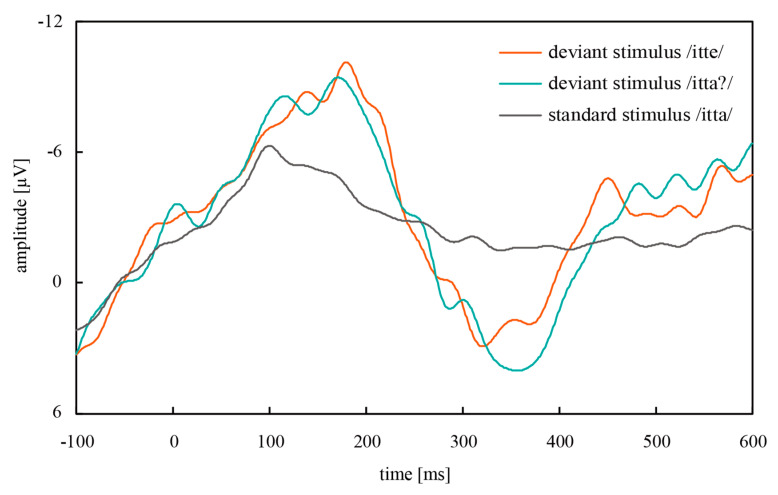
Grand mean waves measured at Cz from the ERPs measured at the parietal region of all participants in the oddball task.

**Figure 10:**
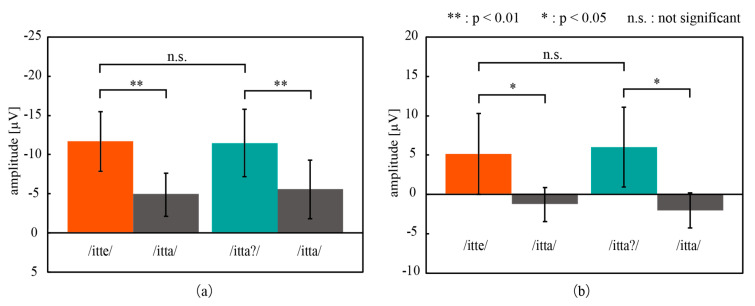
(**a**) MMN and (**b**) P300 obtained in the oddball task consisting of three speech sounds. /itte/ and /itta?/ represent the deviant stimuli, and /itta/ is the standard stimulus. Error bars: the standard deviation.

Figure 9 shows the grand mean waves of ERPs obtained from the parietal region of all participants in the oddball task. A negative deflection, i.e., deflection upward in the figure, appeared around 200 ms after both deviant stimuli, and a positive deflection appeared around 350 ms after both deviant stimuli (itte/itta?). The amplitude of the deflections was significantly different from that for the standard stimulus at the corresponding time, as shown in Figure 10. Based on these results, the negative deflections were considered to be MMN, and the positive deflections were P300. MMN is an ERP that appears as a negative deflection around 150–250 ms after the deviant stimuli and reflects the automatic recognition of the difference in auditory stimuli [20,21]. MMN and P300 were successfully captured by CMEs in this oddball task with three speech sounds.

#### 3.2.2. Simultaneous Measurement of EEG and NIRS

**Figure 11:**
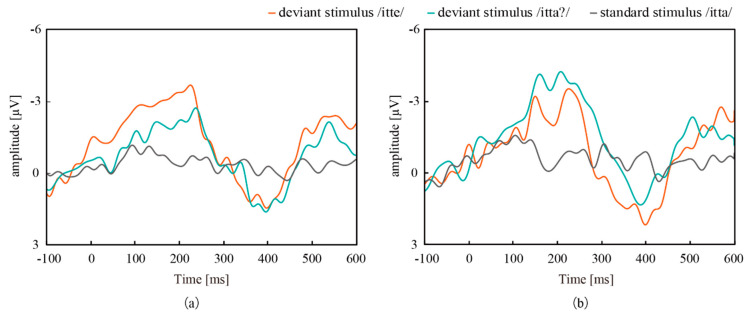
Grand mean waves of EEG-based ERPs measured at (**a**) T3 and (**b**) T4. The ERPs to the deviant stimuli from both measurement sites captured MMN and P300.

**Figure 12:**
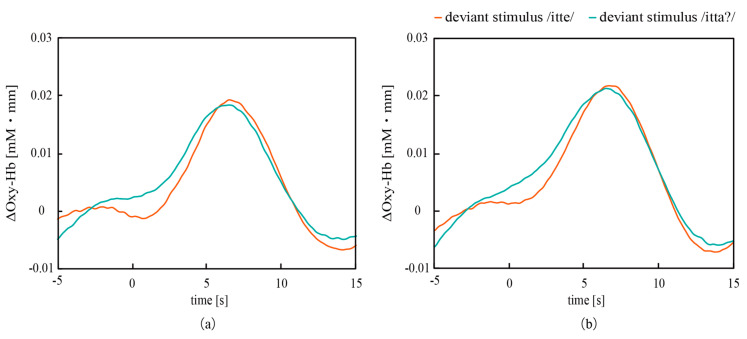
Variation in the concentrations of Oxy-Hb after the deviant stimuli, which were observed in the (**a**) left and (**b**) right auditory areas. Note that EEG has superior temporal resolution to NIRS.

Figure 11 shows the grand mean waves of EEG measured from T3 and T4. The negative deflections and the positive deflections were successfully measured after the deviant stimuli, /itte/ and /itta?/. Since the latencies of these deflections are consistent with those of MMN and P300 obtained in preliminary experiments, these bumps are MMN and P300. Recognition of the vowel and prosody conditions is processed in different parts of the brain and appeared as differences in ERPs from T3 and T4. This laterality will be discussed below. Figure 12 shows the grand mean waves of NIRS measured from the left and right regions, which indicated that the concentrations of Oxy-Hb increased from 3 to 7 s after the deviant stimuli. The results confirmed that EEG measured with both CMEs and NIRS captured the response of the brain to the deviant stimuli, and that EEG showed much a faster response than NIRS.

**Figure 13:**
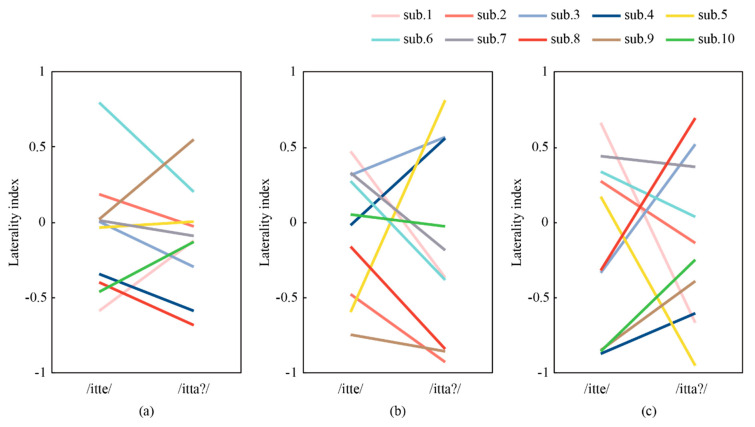
Laterality index (LI) of (**a**) NIRS, (**b**) MMN, and (**c**) P300 for each participant. Six of 10 participants showed greater LI for /itte/ than for /itta?/ as previously reported. Laterality was not captured with EEG.

To assess the laterality quantitatively, we calculated a laterality index (LI=(L−R)/(L+R)) for each participant. With EEG, L and R are the mean amplitude of the grand averaged signal from T3 and T4, respectively, during 5 ms before and after the latency time of MMN and P300 (Table 2). With NIRS, L and R are the mean concentrations of Oxy-Hb of the grand averaged signal from the right and left auditory areas during 2 s before and after the peak of the concentrations in grand mean waves (Table 2). Figure 13a–c shows *LI* of NIRS, MMN, and P300, respectively. In general, a larger *LI* indicates more dependence on the response in the left auditory area. Therefore, ΔLI (LI for /itte/−LI for /itta?/) is more likely to be positive according to recent studies [22,23,28,29].

Figure 13a shows that LI for /itte/ was greater than that for /itta?/ in six of 10 participants (#2, #3, #4, #6, #7, and #8) in the obtained NIRS. According to prior work, 85% of right-handed participants show a positive ΔLI, and 50% of non-right-handed participants show a negative ΔLI [22]. The other four participants may belong to the minor 15% of right-handed individuals or 50% of the non-right handed individuals, although all 10 participants declared that they were right-handed.

Figure 13b shows that the trend of *LI* in MMNs was consistent with four of the six participants (#2, #6, #7, and #8) whose NIRS responses showed a positive ∆*LI*. On the other hand, from Figure 13c, the trend of *LI* in P300 was in agreement with three individuals (#2, #6, and #7); MMN is generated by the auditory cortex and is a response to changes in auditory stimuli [21]. Therefore, the MMNs obtained in this experiment reflect only the MMNs generated in the left and right auditory cortex, which is the same as that of NIRS. On the other hand, P300 is a response to an unexpected stimulus [19] and has been reported to originate from multiple sites including the frontal, parietal, and temporal lobes [30]. Therefore, P300 in the right and left auditory cortex in this experiment is a composite waveform of P300 generated at multiple sites. Therefore, it is likely that the effect of P300 from other sites did not show the same trend as NIRS.

From the above discussion, it can be said that MMN is more likely to detect lateralization by language processing than P300. In addition, comparing the magnitude of the amplitudes of the ERPs in the total additive average waveform of the EEG, the amplitudes of the MMNs were larger in the left side of the brain for phonological differences (/e/, /a/) than for intonation differences (/a?/, /a/), and larger in the right side of the brain for intonation differences (/a?/, /a/) than for phonological differences (/e/, /a/), but not in the P300, suggesting that the MMNs are more likely to detect lateralization by language processing than the P300s.

EEG measurement using CMEs distinguished the automatic recognition (MMN) and the conscious recognition (P300) in real-time due to the fine time resolution. This may lead to new applications in monitoring and intervention using EEG, which can be performed with CMEs that do not require time-consuming preparation. In addition, the good spatial resolution of NIRS measurement and the time resolution of EEG measurement can be combined to further analyze the cognitive activities of the brain.

## 4. Conclusions

The proposed CMEs showed comparable effectiveness to the conventional wet electrodes in ERP measurement without skin preparation. P300 was successfully extracted in the oddball tasks using stimulation with pure tones. With oddball tasks using three words, /itta/, /itte/, and /itta?/, the laterality of the brain activity that originated from how the vowel and prosody were processed was observed in NIRS measurement for six of 10 participants. ERPs that were simultaneously measured with NIRS clearly captured MMN and P300. However, laterality was not conclusively observed in both MMN and P300, although MMN may be more likely to capture the laterality. EEG measured with CMEs may represent the perceptual activities. Further research is mandatory to address the cognitive activities. Given the superior time resolution and fast response of EEG to the events, ERPs containing MMN and P300 will be further explored for new brain-tech applications, to which CMEs that can measure high-quality ERPs without skin preparation or conductive gel will be ready to contribute.

## Figures and Tables

**Table 1:** Correlation between the signals after the deviant and standard stimuli acquired with CMEs and the wet electrodes.

Participant	CME Deviant vs. Wet Deviant	CME Standard vs. Wet Standard	CME Deviant vs. Wet Standard	CME Standard vs. Wet Deviant
1	0.99	0.99	0.44	0.39
2	0.88	0.93	0.11	−0.06
3	0.99	0.94	0.24	0.18
4	1.00	1.00	0.82	0.81
5	0.91	0.44	0.56	−0.27
6	0.72	0.73	0.82	0.12
7	0.99	1.00	−0.05	0.04
8	1.00	0.93	0.55	0.39
9	0.95	0.94	−0.04	0.08
10	0.93	0.92	0.06	−0.03
11	0.98	0.99	0.47	0.52
mean ± SD	0.94 ± 0.08	0.89 ± 0.17	0.36 ± 0.32	0.20 ± 0.31

**Table 2:** The peak of the concentrations, MMN, and P300 in grand mean waves.

ERP	Characteristics	Left		Right	
		/itte/	/itta?/	/itte/	/itta?/
NIRS	ΔOxy-Hb (−)	0.0192	0.0183	0.0218	0.0212
	Latency (s)	6.5	6.5	6.7	6.5
MMN	Amplitude (µV)	−3.69	−2.74	−3.51	−4.25
	Latency (ms)	225	237	225	207
P300	Amplitude (µV)	1.48	1.63	2.16	1.36
	Latency (ms)	397	395	399	388

## Supplementary Materials

The following are available online at https://www.mdpi.com/2072-666X/11/6/556/s1: Audio S1: pure tone sounds for the oddball task.
